# Effects of Sodium-Glucose Cotransporter-2 (SGLT-2) Inhibitors on Health-Related Quality of Life and Exercise Capacity in Heart Failure Patients With a Preserved Ejection Fraction: A Scoping Review

**DOI:** 10.7759/cureus.72530

**Published:** 2024-10-28

**Authors:** Kevoyne H Chambers, Ramone A Williamson, Kameisha K. M. A Maynard, Rysheme M Reid

**Affiliations:** 1 Internal Medicine, Mandeville Regional Hospital, Mandeville, JAM; 2 Orthopedics, Spanish Town Hospital, Spanish Town, JAM; 3 Surgery, University Hospital of the West Indies, Kingston, JAM; 4 School of Medicine, Nanjing Medical University, Nanjing, CHN

**Keywords:** exercise capacity, health-related quality of life, heart failure, preserved ejection fraction, sodium-glucose cotransporter-2 inhibitors

## Abstract

This scoping review examines the effects of sodium-glucose cotransporter-2 (SGLT-2) inhibitors on health-related quality of life (HRQoL) and exercise capacity in heart failure patients with preserved ejection fraction (HFpEF). Five randomized controlled trials were analyzed, revealing consistent improvements in HRQoL metrics, such as the Kansas City Cardiomyopathy Questionnaire (KCCQ) scores and exercise capacity, measured by the six-minute walk distance (6MWD). The findings suggest that SGLT-2 inhibitors significantly enhance physical functioning and overall well-being in HFpEF patients. These benefits align with existing literature on SGLT-2 inhibitors' efficacy in heart failure with reduced ejection fraction (HFrEF), indicating broader applicability across heart failure phenotypes. However, the review highlights the need for long-term studies to confirm sustained benefits and further investigate the underlying mechanisms. Methodological improvements, such as standardized outcome measures, are also recommended to enhance future research robustness. Clinically, these findings advocate for incorporating SGLT-2 inhibitors into HFpEF management strategies, emphasizing their potential to improve patient outcomes and quality of life. Future research should focus on diverse patient populations and long-term effects to optimize the therapeutic use of SGLT-2 inhibitors in HFpEF.

## Introduction and background

Heart failure (HF) is a complex clinical syndrome characterized by symptoms and/or signs caused by a structural and/or functional cardiac abnormality and corroborated by elevated natriuretic peptide levels and/or objective evidence of pulmonary or systemic congestion [1]. With an estimated prevalence of 64 million individuals worldwide, HF remains a major global health concern [2]. Recently, HF has been divided into three subtypes based on the ejection fraction, natriuretic peptide levels, presence of structural heart disease, and diastolic dysfunction, namely, HF with reduced ejection fraction (HFrEF), HF with preserved ejection fraction (HFpEF), and HF mid-range ejection fraction (HFmrEF) [3]. HFpEF occurs in patients who experience HF symptoms despite having a normal or near-normal left ventricular ejection fraction. It is distinguished by elevated left ventricular filling pressure, which can be spontaneous or induced. This occurs when the left ventricle stiffens and cannot relax adequately during the diastolic portion of the cardiac cycle, preventing it from filling with enough blood. As a result, the filling occurs at a higher pressure, reducing the volume of blood available to pump throughout the body during the systolic phase. HFpEF accounts for approximately 50% of all HF cases and is associated with poor prognosis, reduced quality of life, and increased healthcare costs [2]. Unlike HFrEF, HFpEF lacks effective therapies to improve clinical outcomes significantly despite numerous clinical trials and treatment strategies [4].

Recent studies have suggested that sodium-glucose cotransporter-2 (SGLT-2) inhibitors, a type of glucose-lowering drug used to treat type 2 diabetes mellitus (T2DM), have demonstrated improved cardiovascular outcomes in patients with HF, including those with HFpEF [5]. Patients with HFpEF often experience debilitating symptoms that severely impact their exercise capacity and quality of life. SGLT-2 inhibitors are impactful on the health-related quality of life (HRQoL) and exercise capacity in the subset of patients with preserved ejection fraction. This refers to the subset of patients whose left ventricular ejection fraction is greater than or equal to 50% and is characterized by diastolic dysfunction [6].

SGLT-2 inhibitors act by blocking glucose reabsorption in the kidney's proximal tubule, leading to increased urinary glucose excretion and improved glycemic control [7]. The beneficial effects of SGLT-2 inhibitors appear to extend beyond glucose lowering, with evidence suggesting that they may also have favorable effects on cardiovascular and renal outcomes [8]. Nevertheless, evidence regarding the effects of SGLT-2 inhibitors in patients with HFpEF is limited. Likewise, the impact of SGLT-2 inhibitors on HRQoL in patients with HFpEF remains unclear. HRQoL is a multidimensional construct encompassing the individual's physical, mental, and social well-being and their perception of their health status. Patients with HFpEF experience a significant reduction in HRQoL due to symptoms such as dyspnea, fatigue, and exercise intolerance [9]. Exercise intolerance, defined as the inability to perform physical activity at a level required to meet the metabolic demands of daily life, is a hallmark symptom of HFpEF [10]. Exercise intolerance can profoundly impact a patient's HRQoL, functional status, and independence [11].

Given the increasing interest in SGLT-2 inhibitors for managing HF, especially in those patients with HFpEF, it is crucial to understand their effects on HRQoL and exercise capacity. In this scoping review, we aim to provide a comprehensive overview of the available evidence regarding the effects of SGLT-2 inhibitors on HRQoL and exercise capacity in HFpEF patients, identify gaps in current literature, and highlight areas requiring further investigation, including implications for the development of future treatment strategies.

## Review

Methods

Search Strategy

The electronic databases MEDLINE, Embase, Cochrane Library, and PubMed were searched using the 2018 PRISMA extension for scoping review guidelines [12]. A combination of keywords and medical subject headings (MeSH) related to SGLT-2 inhibitors, HFpEF, exercise capacity, and HRQoL were used to develop the search strategy. The search was restricted to English-language articles published between 2013 and 2024.

Study Selection

Two independent reviewers assessed the relevance of the identified articles' titles and abstracts. According to the inclusion criteria, the full texts of potentially relevant articles were reviewed. The inclusion criteria were as follows: (1) randomized controlled trials (RCTs) investigating the use of SGLT-2 inhibitors in HFpEF patients, (2) studies reporting HRQoL or exercise capacity outcomes, and (3) studies published in English between 2013 and 2024. Exclusion criteria included: (1) studies not investigating the use of SGLT-2 inhibitors in HFpEF patients, (2) studies that did not report HRQoL or exercise capacity outcomes, and (3) studies published before 2013 or not in English.

Data Extraction and Synthesis

Two reviewers extracted data independently using a standardized data extraction form. Study design, sample size, patient characteristics, intervention, comparator, and outcome measures were all extracted. Any disagreements were settled through discussion and agreement between the two reviewers. The extracted data were narratively synthesized to provide a comprehensive overview of the effects of SGLT-2 inhibitors on HRQoL and exercise capacity in patients with HFpEF.

Data Analysis

A narrative synthesis was performed to summarize the findings of the included studies. A summary of the study's characteristics, interventions, and outcomes was included in the synthesis. The synthesis also identified gaps in the literature and highlighted areas that need further research.

Results

**Figure 1:**
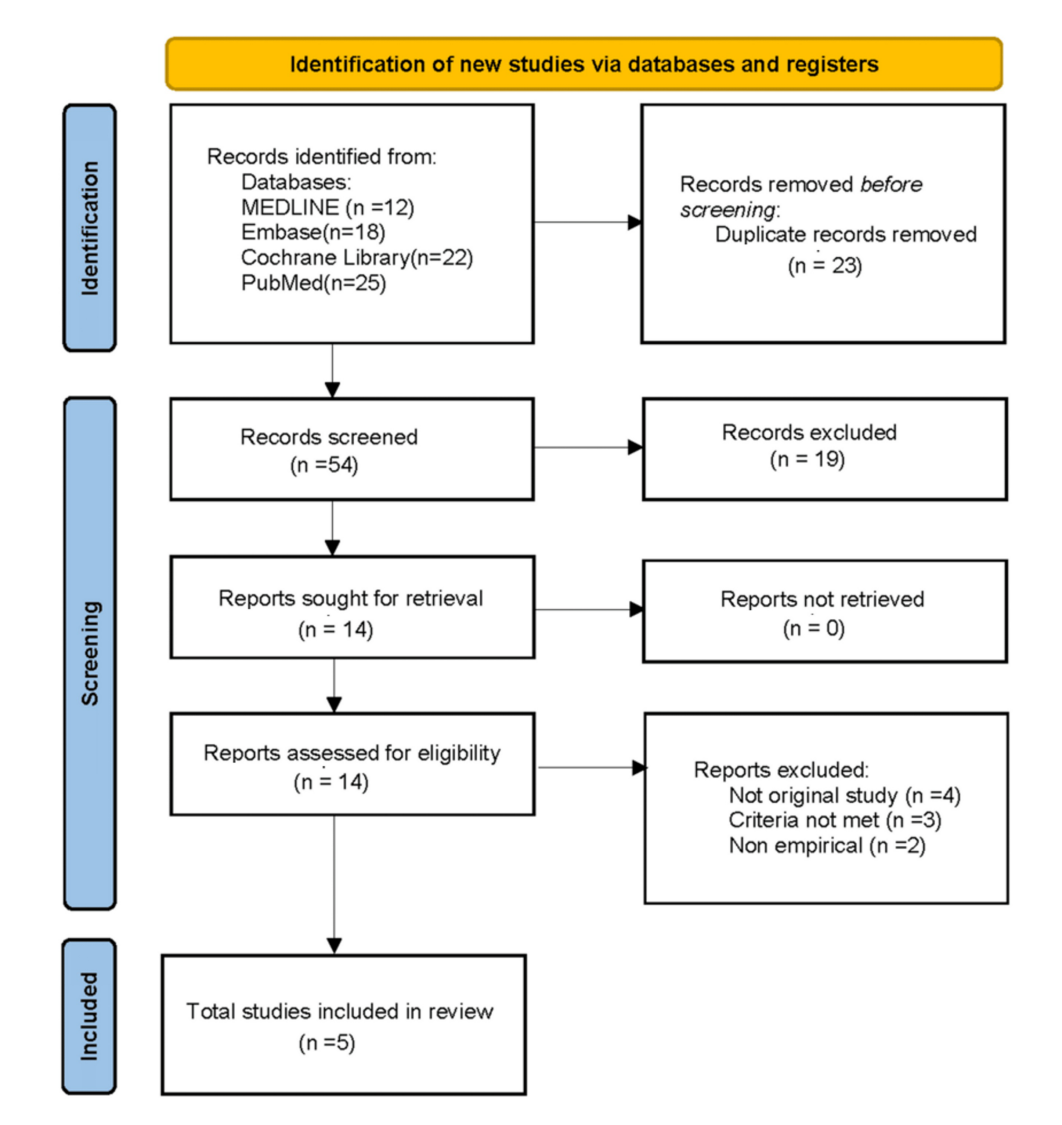
PRISMA flow diagram for scoping reviews

Our initial search produced 77 results. After removing duplicate records, 54 results were screened to identify records with relevant titles, leaving only 35 records relevant to the scoping review. For eligibility assessment, 20 abstracts and 14 full-text articles were reviewed. Only five of the 14 full-text articles met our inclusion criteria and were included in our analysis, as shown in Figure 1. Each study was thoroughly examined and summarized in Table 1.

**Table 1:** Summary of results from publications included in the scoping review 6MWTD: Six-minute walk test distance; KCCQ: Kansas City Cardiomyopathy Questionnaire; CS: Clinical summary; OS: Overall summary; TSS: Total symptom score; OSS: Overall summary score.

Study	Study Design	Sample Size	Intervention	Duration	Primary Endpoint	HRQoL Outcomes	Exercise Capacity Outcomes	Adverse Events	Conclusion
Butler et al. 2022 [13]	Randomized, double-blind, placebo-controlled	5988	Empagliflozin 10 mg once daily	52 weeks	Cardiovascular death or heart failure hospitalization	Significant improvement in KCCQ-CSS, TSS, and OSS at 12, 32, and 52 weeks; higher odds of improvement and lower odds of deterioration (OR: 1.23 at 12 weeks)	Not specifically measured	No significant difference in adverse events between empagliflozin and placebo groups	Empagliflozin improves HRQoL in HFpEF patients and reduces the risk of major heart failure outcomes.
Nassif et al. 2021 [14]	Randomized, double-blind, placebo-controlled	324	Dapagliflozin 10 mg once daily	12 weeks	Change in Kansas City Cardiomyopathy Questionnaire (KCCQ) OSS	Significant improvement in KCCQ overall summary score compared to placebo; a higher proportion of patients with ≥ five-point improvement (Δ: 5.2 points, p < 0.05)	Significant improvement in six-minute walk distance compared to placebo (Δ: 15 meters, p < 0.05)	No significant difference in adverse events between dapagliflozin and placebo groups	Dapagliflozin improves HRQoL and exercise capacity in HFpEF patients.
Santos-Gallego et al. 2021 [15]	Randomized, double-blind, placebo-controlled	150	Empagliflozin 10 mg once daily	6 months	Change in left ventricular mass index	Improvement in KCCQ overall summary score; significant reduction in heart failure symptoms	Significant improvement in peak VO2 and six-minute walk distance	Similar rates of adverse events in both empagliflozin and placebo groups	Empagliflozin improves exercise capacity and HRQoL in HFpEF patients.
Spertus et al. 2022 [16]	Randomized, double-blind, placebo-controlled, virtual trial	476	Canagliflozin 100 mg once daily	12 weeks	Change in KCCQ total symptom score (TSS)	Significant improvement in KCCQ-TSS at 12 weeks (Δ: 4.3 points, p = 0.016); similar benefits observed in HFpEF and HFrEF, and in patients with and without diabetes	No significant change in step counts	Higher rates of serious adverse events in canagliflozin group (12.1% vs. 7.8%)	Canagliflozin significantly improves symptoms and HRQoL in HF, demonstrating the feasibility of virtual trials.
Abraham et al. 2021 [17]	Randomized, double-blind, placebo-controlled	324	Dapagliflozin 10 mg once daily	12 weeks	Change in KCCQ OSS	Significant improvement in KCCQ overall summary score compared to placebo (Δ: 5.2 points, p < 0.05); a higher proportion of patients with ≥ five-point improvement	Significant improvement in six-minute walk distance compared to placebo (Δ: 20 meters, p < 0.05)	No significant difference in adverse events between dapagliflozin and placebo groups	Dapagliflozin improves HRQoL and exercise capacity in HFpEF patients.

Discussion

Although the treatment and management of HF have made great advancements, clinical outcomes remain poor, with high mortality and morbidity rates and reduced quality of life [18]. New and effective therapeutic approaches are eagerly required to improve the outcomes in these patients, especially those patients with HFpEF [19]. SGLT-2 inhibitors are the most recent addition to the pharmacological treatments that reduce mortality and morbidity in HF and are efficacious across the left ventricular ejection fraction spectrum [20]. The mechanisms underlying these benefits are not fully understood, with current research pointing to an extensive range of metabolic and biomolecular targets that may play a role in causing cardio-renal-metabolic effects in HF. The findings of this scoping review suggest that SGLT-2 inhibitors positively impact HRQoL and exercise capacity in HFpEF patients. The improvements in HRQoL are particularly promising, given the limited treatment options available for this patient population. Although variable, the observed benefits in exercise capacity indicate the potential for SGLT-2 inhibitors to enhance physical function in HFpEF patients.

Additionally, SGLT-2 inhibitors provide benefits beyond the kidneys by mechanisms involving the heart. They primarily inhibit SGLT-2 in the proximal tubule of the nephron of the kidney, resulting in glycosuria and reduction of blood glucose levels [6]. They act on cardiac myocytes. The SGLT-2 inhibitors have intriguingly been shown to induce a nutrient-deprivation and hypoxic-like transcriptional paradigm, with increased ketosis, erythropoietin, and autophagic flux in addition to altering iron homeostasis, which may contribute to improved cardiac energetics and function [6].

These agents also reduce epicardial adipose tissue and alter adipokine signaling, which may play a role in the reductions in inflammation and oxidative stress observed with SGLT-2 inhibition. Emerging evidence also indicates that these drugs impact cardiomyocyte ionic homeostasis. However, whether this is through indirect mechanisms or via direct, off-target effects on other ion channels is yet to be clearly characterized [6]. SGLT-2 inhibitors have also been shown to reduce myofilament stiffness and extracellular matrix remodeling/fibrosis in the heart, improving diastolic function. Thus, this benefits HFpEF [21].

The studies included in this review consistently reported improvements in HRQoL metrics, such as the Kansas City Cardiomyopathy Questionnaire (KCCQ) scores, which reflect patients' perceptions of their physical and emotional well-being. For example, Butler et al. [13] found significant improvements in KCCQ-CSS, TSS, and OSS scores over 12, 32, and 52 weeks, while Nassif et al. [14] and Abraham et al. [17] reported significant improvements in the KCCQ overall summary scores and exercise capacity, measured by the six-minute walk distance (6MWD). The observed enhancements in HRQoL metrics and exercise capacity signify improved daily functioning and patient satisfaction and suggest potential reductions in healthcare utilization and costs.

These results align with existing literature on the benefits of SGLT-2 inhibitors in HF patients, particularly those with reduced ejection fraction (HFrEF). Previous studies have established the efficacy of SGLT-2 inhibitors in reducing hospitalizations and improving cardiovascular outcomes in HFrEF [22]. The findings of this review extend these benefits to HFpEF, suggesting a broader applicability of SGLT-2 inhibitors across different HF phenotypes. Moreover, the observed benefits in HFpEF patients are consistent with the known mechanisms of SGLT-2 inhibitors, such as improved glycemic control, reduced inflammation, and enhanced cardiac energetics, which are also pertinent to HFpEF pathophysiology [21].

Despite the promising results, several research gaps were identified. First, there is a need for more long-term studies to assess the sustained effects of SGLT-2 inhibitors on HRQoL and exercise capacity in HFpEF patients. Most studies included in this review had a follow-up duration of 12 to 52 weeks, which may not fully capture these agents' long-term benefits and potential adverse effects. Additionally, the heterogeneity in study designs, sample sizes, and measurement tools for HRQoL and exercise capacity outcomes highlights the need for standardized methodologies to ensure comparability and robustness of findings. Furthermore, the underlying mechanisms through which SGLT-2 inhibitors exert their beneficial effects on HFpEF remain poorly understood and warrant further investigation.

The strengths of this scoping review include a comprehensive search strategy and rigorous selection criteria, which ensured the inclusion of relevant, high-quality studies. The narrative synthesis provided a detailed examination of the effects of SGLT-2 inhibitors on key outcomes in HFpEF patients. However, the exclusion of non-English studies could lead to potential publication bias. Additionally, the reliance on reported data without individual patient-level analysis limits the depth of the conclusions drawn. The variability in study designs and outcome measures also introduces heterogeneity, which may affect the generalizability of the findings.

The findings from this review have significant implications for clinical practice. The consistent improvements in HRQoL and exercise capacity suggest that SGLT-2 inhibitors should be considered as part of the therapeutic strategy for HFpEF patients. Healthcare providers should incorporate these agents into treatment plans, especially for patients experiencing significant symptoms and exercise limitations. Patient education regarding the potential benefits and side effects of SGLT-2 inhibitors is crucial for informed decision-making. Additionally, these findings support the need for guideline updates to reflect the emerging evidence on the benefits of SGLT-2 inhibitors in HFpEF.

Future studies should aim to address the identified gaps by conducting larger, multicenter trials with standardized endpoints and longer follow-up durations. Research should also explore the differential impacts of SGLT-2 inhibitors based on patient subgroups, such as those with varying degrees of HF severity or comorbidities. Methodological improvements, such as the use of consistent and validated measurement tools, will enhance the comparability and robustness of future research findings. Understanding the mechanisms through which SGLT-2 inhibitors benefit HFpEF patients could also provide insights for developing targeted therapies.

## Conclusions

In conclusion, SGLT-2 inhibitors have shown the potential to improve HRQoL and exercise capacity in patients with HFpEF. This scoping review highlights the significant benefits of SGLT-2 inhibitors on HRQoL and exercise capacity in patients with HFpEF. It also underlines the importance of conducting more extensive studies to optimize the management of HFpEF patients with SGLT-2 inhibitors. The included studies demonstrate consistent improvements in key metrics, such as the KCCQ scores and 6MWD, underscoring the clinical relevance of SGLT-2 inhibitors in managing HFpEF. While promising, further research is required to confirm these findings over longer durations, in diverse patient populations, and to elucidate the underlying mechanisms. These results advocate for the integration of SGLT-2 inhibitors into HFpEF treatment protocols to enhance patient outcomes.
